# Minimally Invasive Bowel Cancer Detection through Vibrating Microrobot‐Induced Elastography

**DOI:** 10.1002/aisy.202400926

**Published:** 2025-05-19

**Authors:** Andrew Bickerdike, Jiyuan Tian, Yang Liu, Shyam Prasad

**Affiliations:** ^1^ Exeter Small‐Scale Robotics Laboratory Engineering Department University of Exeter Exeter EX4 4QF UK; ^2^ Department of Gastroenterology Royal Devon University Healthcare NHS Foundation Trust Barrack Road Exeter EX2 5DW UK

**Keywords:** bowel cancers, early detections, elastographies, microrobots, vibrations

## Abstract

Early detection of bowel cancer is crucial for substantially improving patient outcomes, highlighting the need for less invasive diagnostic methods. Herein, an innovative diagnostic application of vibrating microrobots combined with laser speckle contrast imaging (LSCI) to minimally invasively estimate the elasticity of potential tumors and surrounding healthy tissue is proposed. By positioning a vibrating microrobot on tissue surfaces, the resonant frequencies of the resulting vibrations are analyzed to create detailed elasticity maps. These maps reveal tumor margins and provide critical information about the tumor's properties spatially. This approach leverages the high spatio‐temporal resolution and noncontact nature of LSCI to offer a minimally invasive elastography method for potential use in colonoscopy, providing an alternative to complex and time‐consuming biopsy analysis and advancing future cancer diagnostics.

## Graphical abstract

A minimally invasive diagnostic method for bowel cancer detection combines vibrating microrobots and laser speckle contrast imaging to map tissue elasticity. By analyzing vibration‐induced resonance, detailed elasticity maps are produced, clearly differentiating tumors from healthy tissue. This innovative approach offers a promising, biopsy‐free alternative for efficient and accurate tumor characterization during colonoscopy

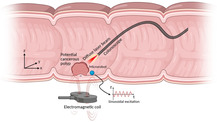

## 1 Introduction

**Figure 1:**
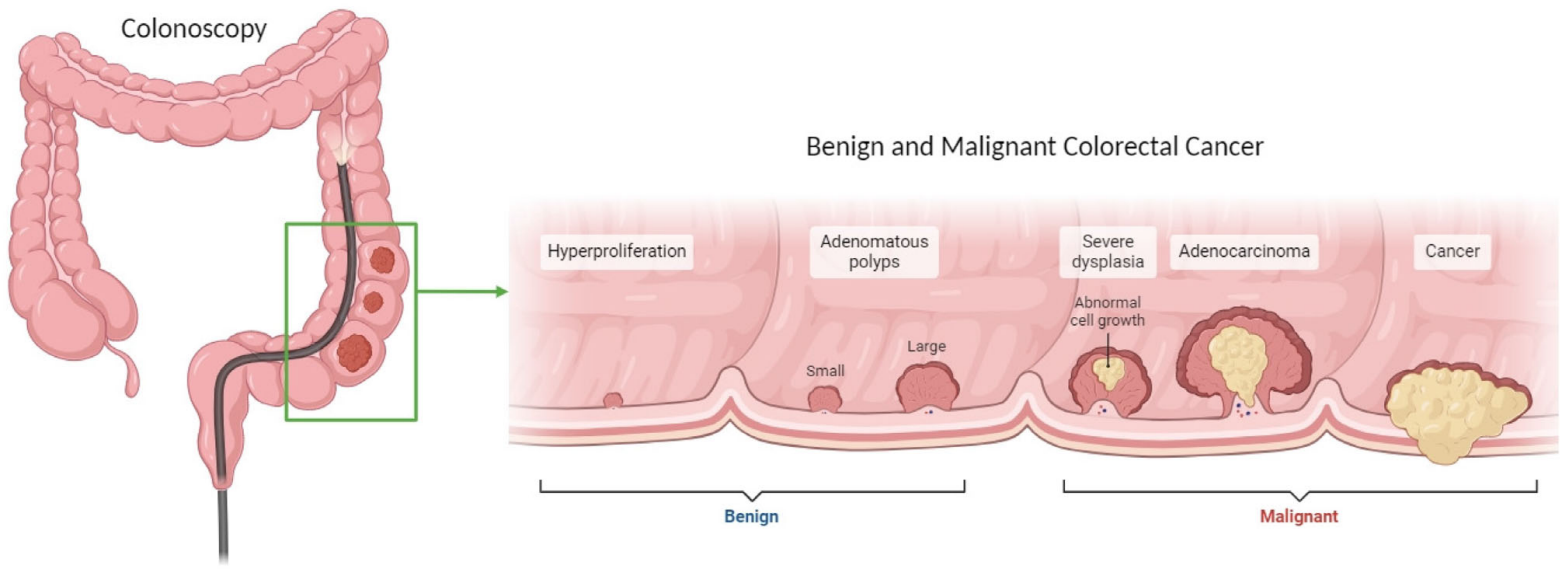
Schematic diagram depicting the progression of bowel cancer from a region of hyperproliferation. As hyperproliferation continues, benign polyps form. The adenomatous polyps undergo dysplastic changes, characterized by abnormal cell growth and loss of normal cellular differentiation. Further genetic mutations cause the dysplastic polyps to transform into early‐stage adenocarcinoma, marked by the invasion of malignant cells into the submucosa, but limited to within the bowel wall. The final stage involves the invasion of tumor cells beyond the bowel wall into surrounding tissue and lymph nodes through blood and lymphatic vessels.

The bowel frequently becomes the site of malignancies that pose significant diagnostic challenges due to their subtle onset and progression.^[^ ^1^ ^]^ The early diagnosis of malignant tumors, as shown in **Figure** **1**, is crucial for effective patient treatment and a favorable prognosis.^[^ ^2^, ^3^ ^]^ Methods such as fecal occult blood tests^[^ ^4^, ^5^ ^]^ and fecal immunochemical tests^[^ ^6^ ^]^ exist as early screening tests to detect blood in the stool. Although being noninvasive, they can yield false results and do not provide detailed information about the tumor. Colonoscopy procedures remain the gold standard for detecting lesions in the colon and rectum. They allow direct visualization of the colon, enabling the detection and removal of polyps immediately. During standard colonoscopies the main way to determine if a polyp is cancerous is by eye and the discretion of the clinician performing the procedure, often with the aid of narrow‐band imaging (NBI), where the detail of surface patterns and the microvasculature of the mucosa is enhanced by viewing the tissue under specific wavelengths of blue and green light.^[^ ^7^, ^8^, ^9^ ^]^ However, NBI has limitations, including its inability to provide detailed information about deeper tissue layers and its dependance on the clinician's experience for accurate interpretation. Therefore, developing a methodology to provide noninvasive, high‐resolution, and reliable diagnostics is crucial. Advancements in medical imaging have highlighted the importance of tissue stiffness as a biomedical marker for distinguishing malignant from benign or healthy tissue. By assessing the mechanical properties of the colon, it is possible to identify diseased tissue and accurately determine the margins of these lesions.^[^ ^10^, ^11^, ^12^ ^]^

To determine the mechanical properties of soft tissue, an elastography method must in some way provide a contrast between different tissue types. Several elastography methods are currently used in clinical settings. Ultrasound elastography utilizes two main methods, strain and shear wave elastography, capable of imaging tissue up to depths of 5 cm. In strain elastography, a transducer applies minimal pressure to the tissues either continuously or repetitively, and the subsequent tissue displacement is tracked between pairs of ultrasound frames before and after compression,^[^ ^13^, ^14^ ^]^ allowing for elastograms to be derived from the change of radio frequency signals before and after transducer compression by using the axial gradient of the displacements. Shear wave elastography operates on the principle that shear waves, generated by a focused ultrasonic beam, travel faster in stiffer tissues.^[^ ^15^ ^]^ By tracking the shear wave speed, it is possible to construct elastograms of tissue stiffness to a high level of detail. Magnetic resonance elastography (MRE) measures micron‐level displacements from externally generated mechanical waves propagating through the tissue. MRE reconstructs an image based on the measured wavelength, relating to the tissue stiffness using MRI scanning.^[^ ^16^ ^]^ Optical coherence elastography (OCE) uses optical coherence tomography (OCT) to map tissue stiffness at a microscopic scale. It applies mechanical stress to tissues, using differences in refractive index to create high‐resolution maps of tissue elasticity^[^ ^17^, ^18^, ^19^, ^20^ ^]^ or track the velocity of propagating waves, calculating material stiffness based on propagation velocity.^[^ ^21^, ^22^, ^23^ ^]^ A benefit of OCE is that en‐face elastograms can be constructed, allowing for the surface elasticity of tissues to be assessed.^[^ ^24^ ^]^

Despite the utility of these methods, traditional elastography techniques face significant challenges when used in a setting to diagnose bowel cancer. The anatomical complexity and need for minimally invasive procedures limit the effectiveness of conventional elastography approaches. Therefore, there is an incentive for novel elastography techniques that can overcome these limitations and provide accurate, noninvasive diagnostics for potential bowel tumors.

We propose a new elastography method utilizing magnetic microrobots and laser speckle contrast imaging (LSCI) as a noninvasive imaging modality to detect and quantify changes in tissue stiffness within the bowel. Previously, other studies have assessed the possibility of using LSCI in some form as an elastography method, to track the propagation of Rayleigh waves on the surface of samples explicitly and using the propagation velocity to determine material stiffness.^[^ ^25^ ^]^ Additionally, LSCI has been utilized as part of a forward scattering experimental setup to track the propagation of shear waves in turbid agar samples to estimate the Young's modulus from shear wave speed.^[^ ^26^, ^27^, ^28^ ^]^ However, these methods require either a sequenced imaging protocol to be timed with a source of mechanical stimulation or high‐speed imaging devices.

**Figure 2:**
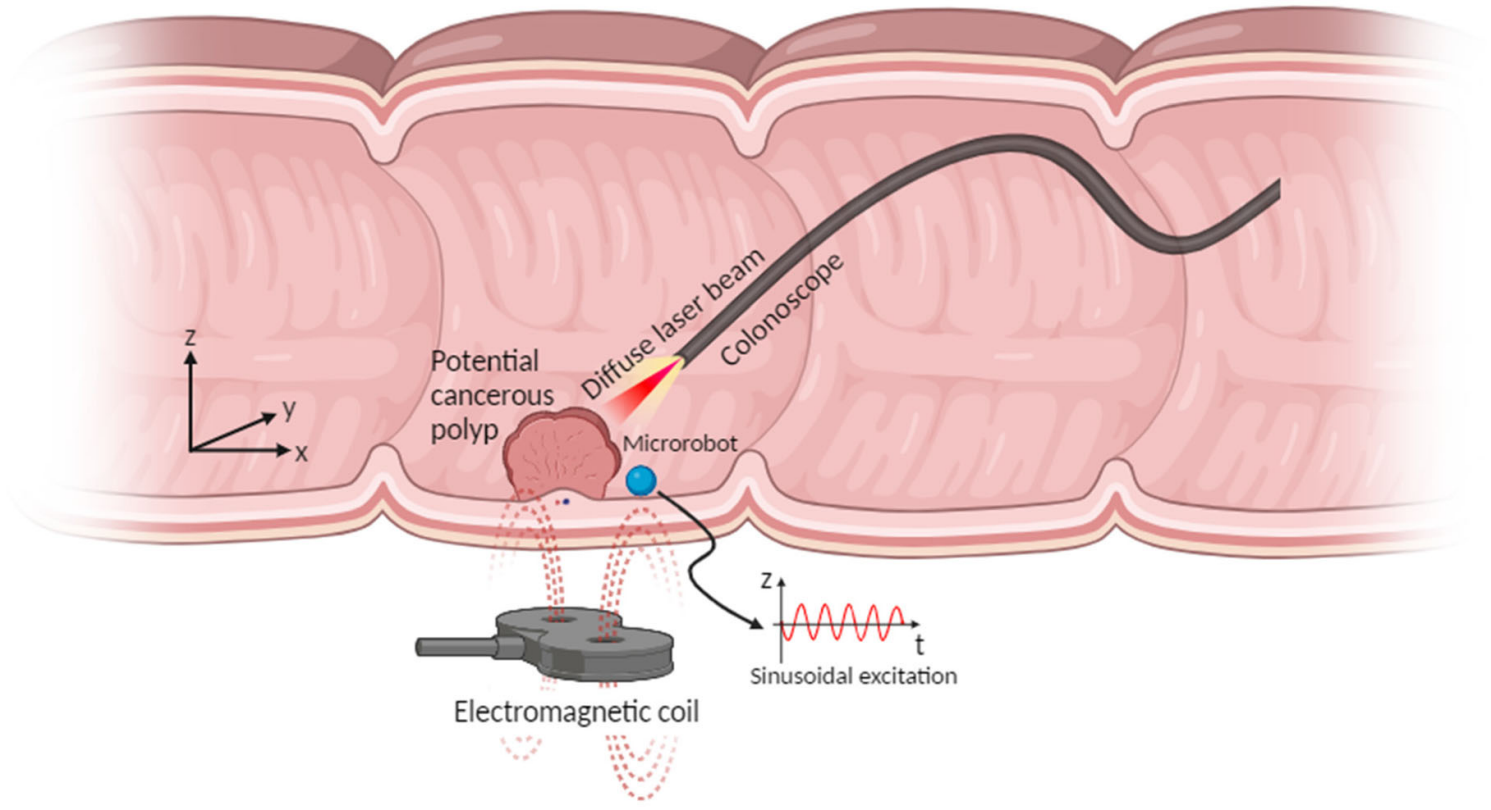
A conceptual procedure showing a microrobot being used in tandem with LSCI for generating elastograms of colon polyps. The schematic presents the electromagnetic coil to actuate the microrobot with a sinusoidal excitation and to position the microrobot on the surface of the colon. The diffuse laser beam projects the speckle pattern onto the surface of the tissue, whilst the camera in the colonoscope records the raw speckle images to be processed into elastograms.

As illustrated in **Figure** **2**, our approach combines the use of magnetic microrobots and LSCI to produce a noncontact, highly sensitive technique capable of optically imaging the mechanical properties of soft tissue without the need for any specialist equipment. In this method, a magnetic microrobot is introduced to the tissue and excited through a range of frequencies using an external magnetic field. When sweeping the frequency range, Rayleigh waves propagate from the contact point of the microrobot on the surface of the tissue, lateral displacement of the retrograde elliptical motion of the surface tissue can be observed using LSCI.^[^ ^29^ ^]^ Based on the assumption that the magnitude of surface displacement measured by LSCI is proportional to the vertical displacement of the vibrating microrobot, the resonant frequency of vibration can be used to calculate the stiffness of different tissue samples. We validated the method across a range of tissue phantoms, designed to replicate similar tissue stiffness through the progression of bowel cancer.^[^ ^30^ ^]^ Additionally, we use this technique to map both the boundaries and stiffness of artificial agar polyps injected into the surface layers of an ex vivo porcine colon. We showed a relation between the increase in material stiffness and the measured resonant frequency of the local material to the microrobot. This technique enables detailed mapping of tissue elasticity, providing a novel tool for characterizing malignant lesions in the bowel.

## 2 Results

### 2.1 Validation of Experimental Methods

**Figure 3:**
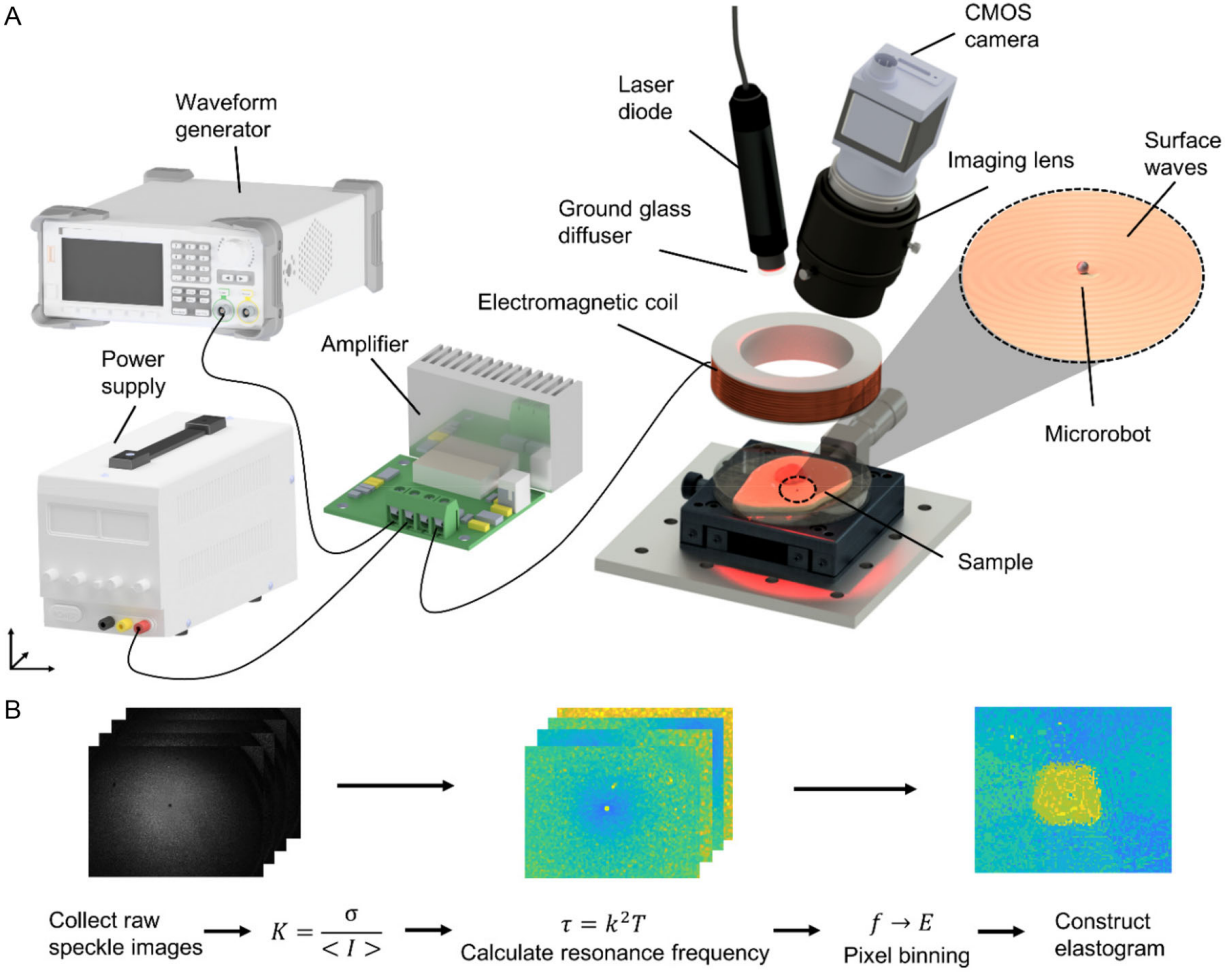
A) Diagram of the experimental rig setup in a backscattering LSCI configuration used to visualize the change in *τ* for different actuation frequencies relating to the magnitude of horizontal displacement of the surface tissue during retrograde motion of propagating Rayleigh waves. B) The processing sequence used to generate elastograms.

**Figure 4:**
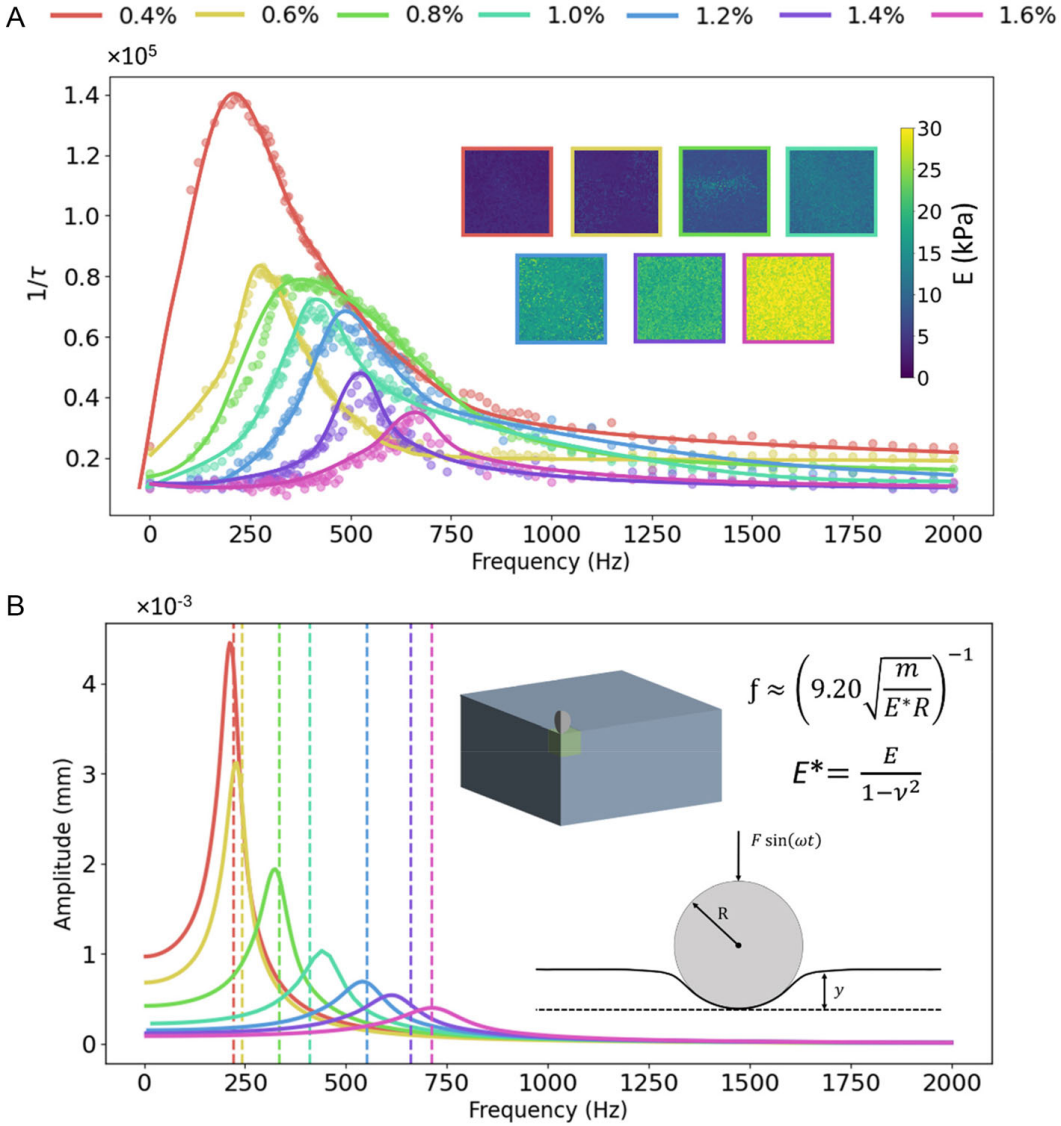
A) Comparison of the resonant curves of the values for 1/*τ* for homogeneous agar phantoms of different wt%. The curves are fitted to average values for each frequency within a 5 × 5 pixel region of interest for each phantom. B) FEA results showing the resonant frequencies of the microrobot vertical displacement and associated resonant frequencies calculated using the mathematical model (vertical dashed lines).

Before we performed the analysis on heterogeneous phantoms and ex vivo samples, we first aimed to validate the accuracy of our method. To do this, we measure the resonant frequencies by performing frequency sweeps from 0 to 2 kHz of a single microrobot vibrating on homogeneous agar phantoms of known stiffness. The microrobot chosen measured a diameter of 500 μm. We used the same microrobot for all the experiments presented in this paper due to the magnetic force being sufficient to generate noticeable surface wave propagation whilst maintaining a size considered microscale. The agar phantoms were constructed from agar powder (Special Ingredients, UK), mixed with water at varying ratios by weight, and added with evaporated milk to simulate the optical scattering properties of soft tissues.^[^ ^31^, ^32^, ^33^ ^]^ The size of each phantom measured 50 × 50 mm. Due to the size and elastic properties of the samples, we assumed that the boundary conditions remained unreflective, meaning the generated Rayleigh waves do not reflect from the edges of the sample.^[^ ^34^ ^]^ To perform the LSCI analysis, we constructed the experimental rig in a backscattering configuration,^[^ ^35^, ^36^ ^]^ shown in **Figure** **3**. The magnetic field gradient at 30 mm from the coil center was measured to be 0.48 T m^−1^. To ensure consistency, the experimental configuration remained the same throughout all experimentation. Sequences of 100 raw images were collected at each frequency step at a framerate of 500 frames per second and an exposure time of 2 ms. An exposure time of 2 ms was chosen to maintain the sensitivity to relative changes in speckle contrast, whilst minimizing the speckle contrast noise.^[^ ^37^ ^]^ The configuration of the lens and the sensor size of the camera allowed raw speckle images to be collected with a field of view of 20 × 20 mm. Once all the raw images were acquired, the aim of the analysis was to measure the values of correlation time, *τ*. Ground truth elastic modulus values for the range of agar samples were previously measured to be between 3 and 35 kPa on a universal testing frame using a procedure of spherical indentation testing (see Supporting Information). The fitted resonant curves of 1/*τ* for different phantoms are shown in **Figure** **4**a, with the softest and stiffest samples exhibiting resonant frequencies of 220 Hz and 710 Hz, respectively. To convert the resonant frequencies to values of elastic modulus, we used a mathematical model^[^ ^38^ ^]^ for a sphere vibrating on an elastic half‐space expressed as

(1) $$f\approx {\left(9.20\sqrt{\frac{m}{E^{*}R}}\right)}^{-1}$$

(2) $$E^{*}=\frac{E}{\left(1\right)-\nu^{2}}$$

where *f* is the resonant frequency, *m* is the mass of the microrobot, *E* ^*^ is the reduced modulus of the phantom, and *R* is the radius of the microrobot. *E* and *ν* are the sample Young's modulus and Poisson ratio, respectively. This model is valid for harmonic vibrations in which the vertical displacement of the sphere is much less than 0.1*R*, a condition that holds throughout all the analyses. *ν* is assumed to be 0.5. Taking the peak 1/*τ* value of the fitted curves and the corresponding values of frequency, we converted the frequencies to elastic modulus and compared them to the ground truth values. With increasing agar percentage, there is an increase in the LSCI measured Young's modulus. LSCI‐derived Young's modulus values were calculated to be 3.22, 3.83, 7.42, 11.28, 20.62, 26.5, and 33.45 kPa for agar samples 0.4, 0.6, 0.8, 1.0, 1.2, 1.4, and 1.6 wt%, respectively. The results from LSCI exhibited strong agreement with the ground truth elasticity measurement values derived from spherical indentation testing, where each sample was tested at four locations, three times, totaling 12 independent measurements per sample, with mean values and standard deviation shown in **Table** **1**. Finite element analysis (FEA) simulations were further used to validate the resonant frequency of the microrobots on the experimental samples. A quarter model based on Hertz contact of a microrobot on an elastic half‐space was set up and executed using the harmonic response method to calculate the resonant frequencies of the vertical displacement of the microrobot. The elastic properties of the samples used in the simulations were matched to the equivalent ground truth Young's modulus values of our phantoms, assuming a density of 1000 kg m^−^^3^ and a frictionless, purely elastic contact. Figure 4b shows the resonant curves from the FEA simulations for each stiffness of the sample. By taking each resonant frequency from the simulations and calculating the Young's modulus using the mathematical model, we validated our method by comparing it to the Young's modulus values originally input into the simulations. The results closely matched the resonant frequencies calculated from the mathematical model using Equations (1) and (2). Additionally, the values derived using LSCI were consistent with both simulations and the mathematical model. While the current analysis neglects viscoelastic damping, inherently present in biological tissues, which may influence the amplitude and quality factor of resonance, the resonant frequency itself remains a reliable indicator of tissue stiffness. The assumption of frictionless elastic contact is therefore justified for our primary clinical aim. Future studies incorporating viscoelasticity could further enhance the accuracy and clinical applicability of this method.

**Table 1:** Comparison of Young's modulus values measured using LSCI and ground truth values (with standard deviations) obtained from indentation testing for agar phantoms of varying concentrations.

Agar sample wt%	LSCI Measured *E* [kPa]	Ground truth *E* [kPa]
0.4	3.22 (±2.8)	3.23 (±0.74)
0.6	3.83 (±1.46)	3.76 (±0.92)
0.8	7.42 (±1.91)	7.05 (±0.52)
1.0	11.28 (±3.41)	12.25 (±2.54)
1.2	20.62 (±6.15)	21.32 (±1.88)
1.4	26.51 (±4.39)	29.81 (±2.78)
1.6	33.45 (±8.74)	35.74 (±3.61)

### 2.2 Heterogeneous Agar Phantoms

**Figure 5:**
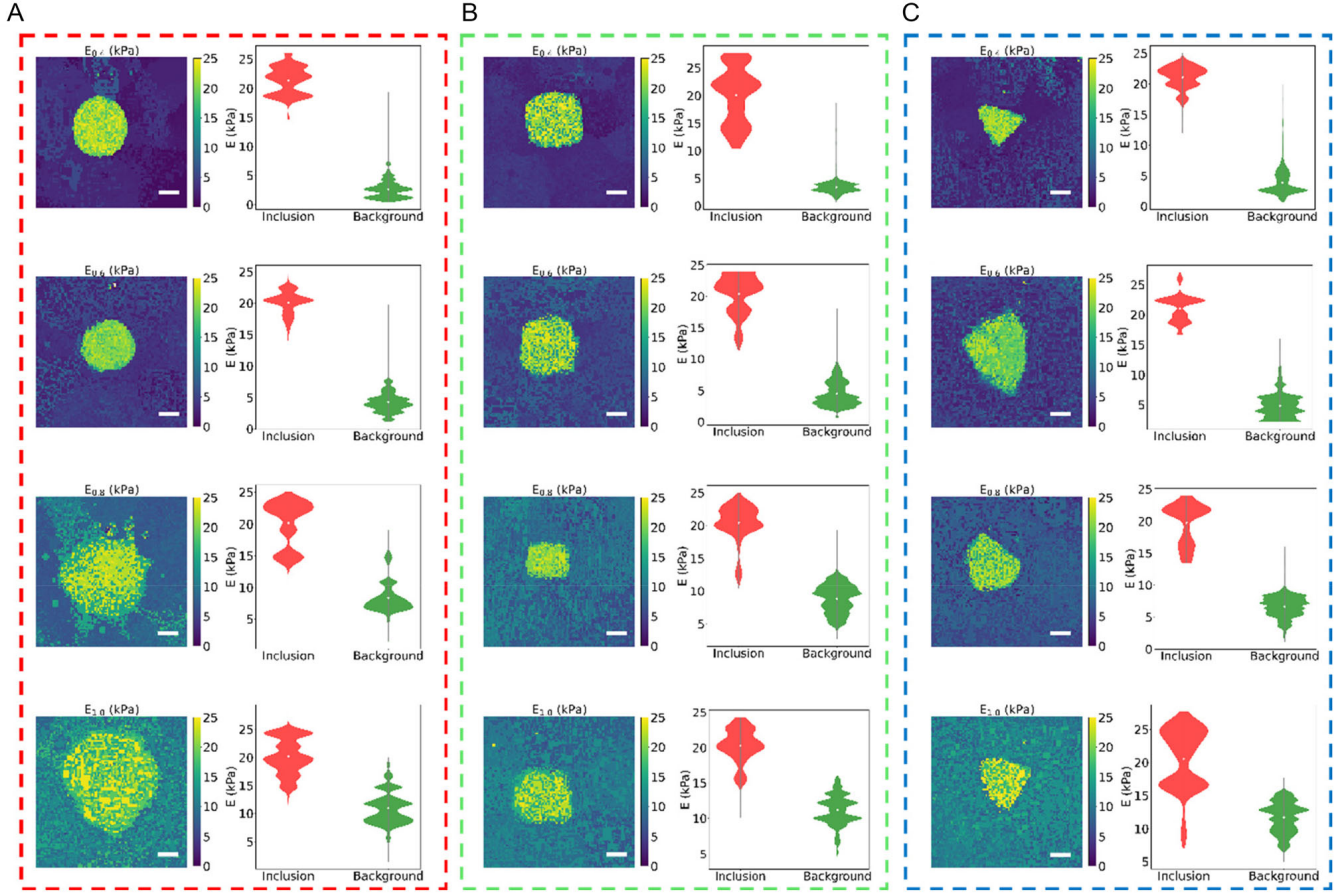
Elastograms and corresponding violin plots of heterogeneous phantoms with A) red box–circular inclusions, B) green box–square inclusions, and C) blue box–triangular inclusions. Red and green violin plots represent the inclusion and background regions, respectively. The elasticity of the background material is varied by agar percentage, with each row representing increasing agar concentrations: Row 1–0.4% agar (0–5 kPa), Row 2–0.6% agar (3–8 kPa), Row 3–0.8% agar (7–10 kPa), and Row 4–1.0% agar (10–13 kPa).

When imaging tumors for diagnosis, it is beneficial to determine the size and shape of a lesion amongst healthy tissue. To replicate this, agar phantoms containing stiff inclusions of defined shapes: circular, square, and triangular, were constructed to investigate the ability of LSCI with vibrating magnetic microrobots to map heterogenous phantoms of varying shape and size. All the inclusions were made from 1.2% agar to provide a consistent stiffness value of ≈22 kPa and were embedded within a background material of varying stiffnesses. A microrobot measuring 500 μm in diameter was positioned by rolling it across the sample surface using an actuation setup consisting of a rotating permanent magnet (NdFeB 25 mm diameter) fixed to a stepper motor, which we could programme for rotational frequency of 5 Hz. The actuation setup allowed precise positioning of the microrobot on the phantom. Frequency sweeps from 0–800 Hz were performed at nine points arranged in a 3 × 3 grid formation, with the central point located at the inclusion's center. This configuration ensured full coverage of the phantom surface, facilitating complete mapping of the wave propagation area. Raw images were collected at each point and processed to produce first, frequency‐encoded elastograms showing the distribution of resonant frequencies for each pixel, and then converted to elastograms showing the surface Young's modulus of the imaging area. Results showed that the boundaries of the inclusions were clearly visible for each shape and followed a consistent mean Young's modulus for each phantom. For different background agar percentages, differences in Young's modulus were evident, with calculated values matching measured ground truth values from indentation testing. Processed elastograms for each shape with varying background material mixtures are presented in **Figure** **5**. The resonant curves for the stiff inclusions exhibited higher frequencies than those of the background material. Notably, triangular and square inclusions had more pronounced edges, showing the method's sensitivity to determine the outline of shapes with sharp and protruding edges. In softer background materials (0.4% and 0.6% agar), the boundaries were more apparent and clearer due to larger differences in resonant frequencies enabling a higher degree of contrast between the inclusion and background material, with the mean background material Young's modulus values ranging from 0 to 5 kPa for 0.4% agar, and 3–8 kPa for 0.6% agar shown in the violin plots. Conversely, in stiffer backgrounds, the boundaries were less clear but still distinguishable. This decreased clarity in stiffer backgrounds can be attributed to the reduced contrast in mechanical impedance between the inclusion and the surrounding matrix, resulting in a less pronounced difference in resonant frequencies. With the mean background material Young's modulus values ranging from 7 to 10 kPa for 0.8% agar, and 10–13 kPa for 1% agar shown in the violin plots. Despite this, the technique effectively distinguished inclusions across various stiffness environments.

### 2.3 Ex Vivo Colon Tissue with Agar Polyps

We focused on ex vivo porcine colon samples, where agar was injected into the mucosa to create artificial polyps of varying stiffness by altering the agar concentration from 0.4 to 1.6 wt%. Following the same protocol as before, we tested nine distinct points on each sample and positioned the microrobot onto the desired testing positions using the rotating magnetic field setup. Frequency sweeps were performed from 0 to 800 Hz in 5 Hz steps, with 100 photographs taken at each frequency step at an acquisition rate of 500 frames per second and an exposure time of 2 ms. Completing full frequency sweeps for each microrobot position on the tissue of interest and constructing the elastogram took 8 mins. When considering the removal of potentially early‐stage polyps, clinicians often spend a considerable amount of time assessing if removal during the endoscopic procedure is appropriate.

When processing the raw speckle images, greater acoustic attenuation was observed in the colon tissue as compared to the agar phantoms, evidenced by the smaller visible area of surface vibrations around the microrobot. The elastograms showed that the 0.4% agar polyp was not clearly distinguishable due to similar Young's modulus values as the colon tissue, with mean values of the polyp and colon tissue measuring 3.05 and 3.33 kPa, respectively. At a 0.6% agar concentration, the polyp outline became more apparent but still exhibited similar stiffness to the surrounding tissue, with the polyp having a mean Young's modulus of 3.33 kPa compared to the colon tissue's 3.01 kPa.

The 0.8% agar polyp displayed a well‐defined shape with stiffness estimation accurately matching the ground truth value, showing a mean Young's modulus of 7.46 kPa, significantly higher than the surrounding colon tissue. For concentrations of 1.0% and above, the polyp boundaries were distinctly visible, with mean Young's modulus values of 12.87 kPa for 1.0% agar and 21.08 kPa for 1.2% agar, indicating a clear differentiation from the surrounding colon tissue. At 1.4% agar concentration, the polyp demonstrated a mean Young's modulus of 29.55 kPa, while at 1.6% agar, the mean value was measured as 38.12 kPa, showing the greatest contrast in stiffness to the colon tissue.

**Figure 6:**
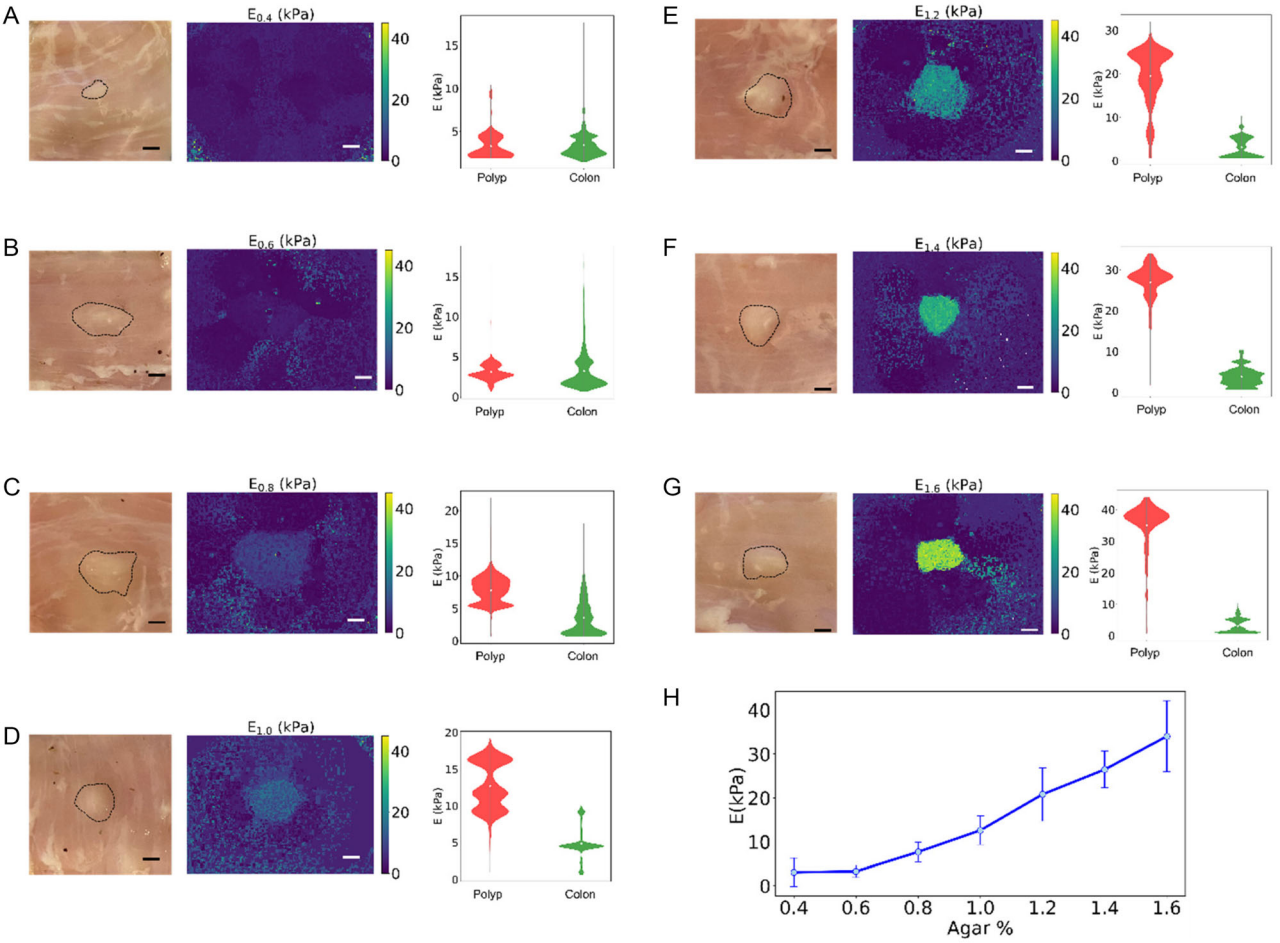
White light and processed elastograms with corresponding violin plots showing the distribution of *E* values within and outside the boundary of artificial agar polyps injected into the mucosa of an ex vivo porcine colon. Polyp elasticity varies with agar wt%: A) 0.4%, B) 0.6%, C) 0.8%, D) 1.0%, E) 1.2%, F) 1.4%, G) 1.6%, and H) Mean polyp stiffness values for different polyp agar wt%. The error bars represent the ± standard deviation of *E* values within the polyp boundaries. All scale bars represent 5 mm.

**Figure** **6** presents white light images with marked polyp boundaries, alongside elastograms that highlight these boundaries. The violin plots illustrate the distribution and variation of stiffness values measured across different agar concentrations. Each violin plot shows the full distribution of measured elasticity data within the boundaries of the polyp, with wider sections indicating a higher frequency of observations at those stiffness values. The mean elasticityhert is marked by a point within each plot, providing a clear indication of the central stiffness tendency at each agar concentration. For the 0.4% agar concentration, the distribution of polyp values matched closely and overlapped with those of the colon tissue, whereas the 0.6% agar concentration showed a broader distribution with a shift towards higher values in the colon tissue. The 0.8% agar polyp violin plot displayed an apparent difference in shape and value distribution from the plot of the surrounding colon tissue, with mean values of 7.32 kPa and 3.22 kPa, respectively. The 1.0% and 1.2% agar concentrations showed even broader distributions, with the bulk of values ranging from 7.2 to 18.0 kPa for 1.0% agar and from 2 to 28.5 kPa for 1.2% agar, likely due to tissue possessing a more heterogeneous structure around where the polyp was injected. The 1.4% and 1.6% agar concentration polyps exhibited mean values of 27 kPa and 34 kPa, respectively, with the corresponding violin plots showing a smaller range of values, with the majority of the values ranging between 20 and 35 kPa for the 1.4% agar polyps, and between 30 kPa and 35 kPa for the 1.6% agar polyps.

## 3 Conclusion

The developed method offers a noninvasive approach to probing the elasticity of soft tissues, a crucial capability for identifying tumor margins during colon procedures. This innovative platform generates detailed elasticity maps of soft tissue surfaces, encompassing both phantoms and ex vivo samples with implanted polyps of varying stiffnesses. The main advantage of this system is its minimal invasiveness, as only a single microrobot interacts with the tissue, which can be precisely controlled externally by a configuration of permanent magnets for positioning and electromagnetic coils for vibration. The system's simplicity is underscored by its reliance on resonant frequency analysis to determine elasticity, employing a straightforward mathematical model to convert between resonant frequency and elastic modulus. Due to the nature of Rayleigh waves, the penetration depth is generally considered to be limited to one wavelength. This functionality could be valuable during colonoscopies, where assessing the stiffness of polyps can indicate potential malignancy, and in surgical contexts, where accurately delineating tumor margins is essential to ensure the complete excision of cancerous tissue. While the model assumes tissue uniformity to enable practical real‐time assessment, this simplification is reasonable for clinical contexts, particularly for early‐stage tumors, such as in colon cancer, typically originate at the surface. Additionally, changes in superficial elasticity are clinically relevant. Stiffness alterations at the surface of polyps or tumors serve as early indicators of malignancy, and detecting these during colonoscopies or surgeries aids in guiding treatment decisions. The spatial resolution of the system is dependent on both the lens and the pixel resolution of the camera sensor. In our experiments, the resolution was observed to be on the millimeter scale, which is adequate for detecting small colon polyps, typically ranging from 2 to 10 mm in size. This level of resolution is sufficient to capture clinically relevant stiffness variations at the surface of the tissue, ensuring that even small polyps can be effectively detected and analyzed during procedures.

Compared to existing clinical and investigational imaging modalities such as conventional colonoscopy, ultrasound elastography, and OCT, our proposed microrobot‐based elastography technique provides distinct advantages. Conventional colonoscopy relies primarily on visual inspection, making it highly operator‐dependent and potentially limited in detecting subtle biomechanical tissue changes. Ultrasound elastography offers depth‐resolved elasticity measurements but generally cannot produce high‐resolution surface maps (en‐face imaging) and may not clearly visualize superficial lesions. OCT provides excellent depth resolution but typically has a restricted imaging field (millimeters), complicating the comprehensive imaging of larger polyps. In contrast, our method provides quantitative, operator‐independent elastography with a relatively large surface imaging area (≈30 mm × 30 mm), enabling effective characterization of larger polyps or tissue regions. Its en‐face imaging capability clearly visualizes spatial stiffness variations across tissue surfaces, enhancing detection of subtle mechanical changes linked to pathological states. However, unlike OCT and ultrasound elastography, our technique lacks depth resolution, restricting analysis primarily to surface or near‐surface layers. In current clinical practice, clinicians typically rely on biopsy to definitively diagnose suspected tumors or abnormal tissues.

The proposed microrobot‐based elastography technique is not intended to replace biopsy entirely, but rather to serve as a complementary tool during screening and surgical procedures. Its primary clinical utility would be to rapidly and noninvasively assess the stiffness of polyps or suspicious tissue areas during colonoscopy, thereby aiding clinicians in identifying potentially malignant lesions requiring biopsy or surgical intervention. This method would be most effectively deployed in scenarios involving screening colonoscopies, enabling real‐time characterization of polyps to improve clinical decision‐making and reduce unnecessary biopsies. Additionally, during surgical resections, this technique could enhance the delineation of tumor margins, ensuring complete removal of malignant tissues while preserving surrounding healthy structures.

The system utilizes a microrobot positioned on the surface of soft tissue, subject to sinusoidal forcing from an external electromagnetic coil. This vibration generates propagating Rayleigh waves, causing surface microrobots to move in a retrograde elliptical motion proportional to the microrobot's displacement. The displacement of these surface microrobots, which act as scattering microrobots, is imaged using LSCI. This technique allows us to measure the relative displacements between tissues of different stiffnesses with high sensitivity through a sweep of frequencies, as the blurring of laser speckle contrast values is directly influenced by scatterer displacements. A Complementary Metal‐Oxide‐Semiconductor camera captures the laser speckle patterns. A key innovation of our method is that it does not require high frame rate acquisition systems like OCE or ultrasound elastography. Instead, it relies on obtaining resonant curves, making the approach both low‐cost and simple while maintaining the ability to provide quantitative results.

We validated our system's performance against samples with known stiffness values measured through indentation testing. Our method achieved results similar to the ground truth values, demonstrating its accuracy and validity. FEA simulations further supported these findings. For phantoms with inclusions, the system successfully delineated the outlines of the inclusions and accurately calculated their elasticity. In the experiments where we injected agar to form artificial polyps in the top layer of porcine colon tissue, despite the higher attenuation of waves due to increased viscosity, the method effectively identified polyp margins, particularly with stiffer polyps where the difference in stiffness made the boundaries more visible.

The applicability of this method in clinical settings is significant, as ex vivo porcine colon closely mimics the human colon in terms of stiffness and viscosity. Utilizing only a single microrobot that can be externally controlled, the system could be integrated with a laser speckle setup on a colonoscope or laparoscope.^[^ ^39^, ^40^, ^41^, ^42^ ^]^ One potential solution is the use of optical fibers coupled with lasers and a chip on tip camera, which would allow for real‐time imaging and elasticity measurement during the procedure. This setup could be integrated into a small, self‐contained module that fits within the instrument channel of a standard colonoscope or endoscope. This approach eliminates the need for direct tissue contact with probes, enhancing patient comfort and allowing for real‐time analysis during colonoscopies or surgeries. Additionally, the deployment of multiple microrobots autonomously navigating to polyps of interest within the bowel could streamline the procedure.

Maintaining stability between the imaging sensor and the sample is essential in LSCI to prevent speckle blur, which reduces image quality and accuracy. This stability becomes particularly challenging when considering clinical applications involving the colon, where inherent physiological motions, such as peristaltic contractions and breathing movements, introduce significant motion artifacts. Colon peristalsis, the rhythmic contraction of intestinal muscles, naturally generates movements that can blur speckle patterns and complicate accurate elasticity measurement. Additionally, respiratory motion further exacerbates this issue by introducing repetitive displacement of the abdominal and pelvic regions. Although existing methods have been developed to minimize sensor‐related movements in LSCI,^[^ ^43^ ^]^ addressing tissue or organ‐level motion requires additional consideration. Future strategies could include motion compensation algorithms, real‐time tracking, or adaptive stabilization techniques already established in endoscopic imaging modalities currently being used in clinical practice. Another promising extension involves incorporating real‐time data processing to enable instantaneous elasticity imaging and boundary detection,^[^ ^44^, ^45^ ^]^ analogous to how fluorescence lifetime imaging^[^ ^46^ ^]^ and NBI have been successfully integrated into colonoscopy procedures.

## 4 Experimental Section

4.1

4.1.1

##### LSCI

The dynamic changes in two‐dimensional laser speckle patterns observed on the surface of the sample related to the underlying dynamics of scattering particles being studied. Speckle contrast *K* was defined by

(3) $$K=\frac{\sigma }{<I>}$$

where *σ* and <I> are the standard deviations and mean intensity within a defined pixel kernel in the spatio‐temporal domain, respectively.^[^ ^37^, ^47^, ^48^ ^]^ Speckle contrast is an averaged quantity dependent on the exposure time *T*. However, speckle contrast values only provided a qualitative indication of the degree of scattering particle motion and do not give a quantitative measure of the displacement. To ascertain a measure of displacement on the surface of the sample, we had established a relationship between the speckle contrast value and the correlation time constant *τ* of the speckle autocorrelation function.^[^ ^49^ ^]^

The square of the speckle contrast bears a direct relationship to the normalized electric field autocorrelation function represented as *g*_1_(*τ*)

(4) $$K^{2}=\frac{\sigma^{2}}{{<I>}^{2}}=\frac{2\beta }{T}\int_{0}^{T}g_{1}^{2}(\tau )\left(1-\frac{\tau }{T}\right)dt$$

where *β* is related to the combined effects of speckle averaging, depolarization, and noise. This representation of *K* enables the use of autocorrelation functions $g_{1}\left(\tau \right)\;$to estimate the correlation time *τ* for the speckles. Using the form of $g_{1}\left(\tau \right)=\exp\left(-T/\tau \right)$for the normalized autocorrelation function and inserting it into Equation (4), it is possible to derive the relationship between speckle contrast *K* and the decorrelation time *τ*

(5) $$K^{2}\left(T,\tau \right)=\beta \left[\frac{e^{-2x}-1+2x}{2x^{2}}\right]$$

Where *x* = *T*/*τ*. Therefore, by collecting sequences of images covering a range of *T* values, it is possible to solve for *τ* by implementing a fitting procedure for *K* ^2^ versus *T* using Equation (5), in addition to estimating *β*.^[^ ^48^, ^49^, ^50^ ^]^

To reduce processing time, we used a simplified relationship^[^ ^51^ ^]^ to calculate *τ* by using

(6) $$\frac{1}{\tau }=\frac{1}{K^{2}\cdot T}$$

and applied this technique to analyze frequency sweeps of our magnetic microrobot vibrating on the surface of an elastic medium. At each frequency, we computed the correlation time constant *τ* across the field of view. The vertical oscillations of the microrobot acted as a point source for Rayleigh waves, which propagate across the surface of the material. Due to the elliptical nature of Rayleigh wave motion, the vertical displacement of the particle was coupled to horizontal displacements of the tissue surface. Thus, the vertical vibration amplitude of the microrobot serves as an indirect indicator of the resulting surface displacement. At resonant frequencies, the Rayleigh waves produced the largest displacements in the tissue, and correspondingly, the microrobot undergoes the most sustained vertical vibration. This results in the fastest speckle decorrelation, or equivalently, the maximum value of 1/*τ* in the speckle pattern. By plotting 1/*τ* as a function of frequency, we identifyed the resonant frequency for each pixel as the frequency at which 1/*τ* reached its maximum. This process is repeated for each spatial position of the microrobot across the sample. The result was a pixel‐wise map of resonant frequencies, which were then converted to elastic modulus using a mathematical model in Equation (1), providing spatially resolved elasticity estimates. Data processing was performed using MATLAB (version 2023b, MathWorks, Inc.). Speckle contrast values, represented as *K* ^2^, were calculated for each image at each frequency using a pixel kernel size of seven, then averaged. Equation (3) was used to compute *K* ^2^ for a given exposure time (*T*). To improve signal‐to‐noise ratio and computational speed, 2 × 2 pixel binning was applied to the *K* ^2^ maps. A frequency–1/*τ* curve was then constructed at each pixel, and a resonant frequency was extracted as the peak of this curve. Elastograms of resonant frequency and corresponding elastic modulus were calculated pixel‐by‐pixel and displayed as images. An algorithm was used to combine data from all tested microrobot positions by aggregating neighboring 2 × 2 binned *τ* values. The maximum 1/*τ* value across overlapping positions was selected to represent the strongest resonance, ensuring optimal spatial coverage and signal strength. This procedure results in detailed spatial maps of the sample's mechanical properties, enabling quantitative analysis of tissue elasticity.

##### Image Acquisition

A high‐speed camera (640 × 480 pixels, 4.8 μm pixel size, Basler ace acA640–750 um, Germany) was used to image the speckle patterns through a 50 mm lens with a *f*‐number of 2.8. The speckle size was found proportional to the *f*‐number of the lens; therefore, the Nyquist criterion of speckle size was fulfilled with the speckle size being at least twice the size of the camera pixels. A polarization filter was placed in front of the imaging lens to eliminate artifacts arising from reflected light. The distance between the sample and the lens was 100 mm and remained constant through all the experimentation.

A waveform generator connected to a computer and the electromagnetic coil enabled a programmed frequency sweep from 0 to 2 kHz. 100 frames were obtained at an acquisition rate of 500 frames per second, and varying exposure time from 500 μs to 2 ms for each frequency step, and saved to the computer for post‐processing. The samples were radiated by coherent light from a stabilized laser diode module (633 nm, 5 mW, Quartron, USA). The light was expanded onto the sample by placing a ground glass optical diffuser (Edmund Optics, USA) in front of the diode, resulting in an illuminated area of 12.5 cm^2^ and an irradiance of approximately 4 W m^−2^, well below the maximum permissible power density for human skin.^[^ ^52^ ^]^ The illumination light was modulated to ensure the intensity of the captured images remained the same for different exposure times.

##### FEA Simulations

3D harmonic response analyzes were set up using the commercial software ANSYS Mechanical (ANSYS Inc, USA) to determine the resonant frequencies of the microrobot vibrating on an elastic half‐space to represent the sample. The diameter of the microrobot was modeled to be 500 μm, and the density of the microrobot was set to 4000 kg m^−3^. The half‐space was assumed to be a linear elastic material, for each simulation, we set the Young's modulus to the measured experimental value corresponding to the associated agar percentage, and a uniform density of 1000 kg m^−3^. A quarter model with frictionless support boundary conditions on the cross sections was used to model the system symmetry appropriately. A fixed support boundary condition was applied to the base of the half‐space, and the force was applied from the center of the microrobot. Simulations for the resonant frequencies of the microrobot on the elastic half‐space were performed for frequencies ranging from 0 to 2 kHz. The results were found to be a good match with experimental findings.

##### Microrobot Fabrication

Each microrobot was composed of a cured mixture of elastomer and Neodymium (NdFeB) ferromagnetic powder. The polydimethylsiloxane (PDMS) elastomer (SLYGARD 184, Dow Corning, USA) base and curing agents were mixed in a 10:1 ratio by weight, chosen for its favorable curing profile and elastic properties. The NdFeB magnetic powder (average diameter 5 μm, MQP‐15‐9HD, Neo Magnequench, Singapore) was then mixed into the uncured elastomer at a ratio of 2:1 particle to elastomer by weight by hand for 10 mins. Following this, the elastomer‐particle mixture was transferred to a vacuum oven for degassing, where it was subjected to vacuum conditions of −0.09 MPa for 30 mins to remove any entrapped air bubbles. Subsequently, the degassed mixture was heated at 70 °C for 15 mins in the vacuum oven to partially cure the elastomer and increase its viscosity, making it more manageable for droplet production. An aqueous solution was prepared by mixing 0.5 mL of Triton X‐100 surfactant with 25 mL of deionized water, ensuring the surfactant was completely dissolved to stabilize the droplet size of the elastomer‐particle mixture when added. Using a pipette, 1 mL of the elastomer‐particle mixture was added dropwise to the stirring aqueous solution. This dropwise addition facilitated the formation of uniform microparticles. The mixture was stirred at 1000 rpm and kept at 70 °C for 2.5 hrs to ensure complete curing of the PDMS elastomer, thereby encapsulating the NdFeB magnetic particles within the solidified PDMS. After the curing process, the solution was allowed to cool to room temperature, and the microparticles were collected manually using a pair of needle tweezers and imaged through a microscope. The collected microparticles were then washed thoroughly with deionized water to remove any residual material. They were spread thinly on a clean microscope slide and left to dry at room temperature. Once dry, the magnetic microparticles were collected and stored in a clean petri dish for subsequent size characterization. To characterize the microrobots, we took a random selection of microparticles from the petri dish and imaged them using an upright benchtop microscope.

##### Sample Preparation

Samples were prepared using a mixture of agar, deionized water, and evaporated milk. The powdered agar was mixed with deionized water by weight to create solutions with concentrations of 0.4, 0.6, 0.8, 1.0, 1.2, 1.4, and 1.6 wt%. To each mixture, we added 10 wt% of evaporated milk to provide the samples with optical scattering properties appropriate to the colon tissue.^[^ ^53^ ^]^ The liquid mixtures were mixed using a magnetic stirrer and hot plate for 15 mins at 90 °C. Homogeneous samples were prepared by pouring the mixture directly into petri dishes. For samples containing inclusions, the liquid mixture was first cast into 3D printed molds, allowed to solidify, and then placed in a petri dish. The background material was subsequently poured around it within the petri dish to ensure uniform distribution. Once poured, all agar phantoms were left to set at room temperature for 50 mins.

For the colon samples, we used frozen porcine intestine, which we defrosted overnight. Once thawed, the colon samples were cut into sections, and artificial polyps of varying stiffness were injected into them using the prepared agar mixtures. To prevent dehydration and to maintain the mechanical properties of the colon tissue, the samples were kept in a water bath when not being tested.

##### Generated Magnetic Field

The magnetic field and gradient were measured. Using a z‐axis linear optical stage with a hall probe attached, the magnetic field strength readings were measured at increasing distances from the center of the magnetic coil and compared to the theoretical prediction of the magnetic field strength at a distance from the coil center.

(7) $$B\left(z\right)=\frac{\mu_{0}NIa^{2}}{2{\left(a^{2}+z^{2}\right)}^{\frac{3}{2}}}$$

Where $\mu_{0}$ is the permeability of free space, *N* is the number of turns in the coil, *a* is the radius of the coil, *I* is the coil current, and *z* is the axial distance from the center of the coil. The gradient field was calculated by differentiating the values of *B* with respect to axial distance. The measured *B* field values and calculated gradient field values match the theoretically derived values well. The maximum gradient field value of 1 T m^−1^ was present 12.5 mm from the center of the coil.

## Conflict of Interest

The authors declare no conflict of interest.

## Author Contributions

**Andrew Bickerdike**: conceptualization (lead); data curation (lead); formal analysis (lead); investigation (lead); methodology (lead); software (lead); validation (lead); visualization (lead); writing—original draft (lead); writing—review and editing (lead). **Jiyuan Tian**: data curation (supporting); investigation (supporting); methodology (supporting); software (supporting); validation (supporting); visualization (supporting); writing—original draft (supporting). **Yang Liu**: conceptualization (lead); funding acquisition (lead); methodology (supporting); project administration (lead); resources (lead); supervision (lead); writing—review and editing (equal). **Shyam Prasad**: supervision (equal); writing—review and editing (equal).

## Supporting information

Supplementary Material

## Data Availability

The data that support the findings of this study are available from the corresponding author upon reasonable request.
