# Supplementary material for: Minimally Invasive Bowel Cancer Detection through Vibrating Microrobot‐Induced Elastography
Source: Adv Intell Syst. 2025 May 19;7(8):2400926. doi: 10.1002/aisy.202400926 (PMC12370167; doi:10.1002/aisy.202400926)
Supplement: Supplementary file 1 — Supplementary Material [file AISY-7-0-s001.zip › AISY.202400926-sup-0001-supdata-S1/Supporting Information.pdf]

Supporting Information

Title Laser speckle contrast imaging based elastography using magnetic microrobots

Andrew Bickerdike,[1] Jiyuan Tian,[1] Yang Liu,[1] Shyam Prassad[2]*

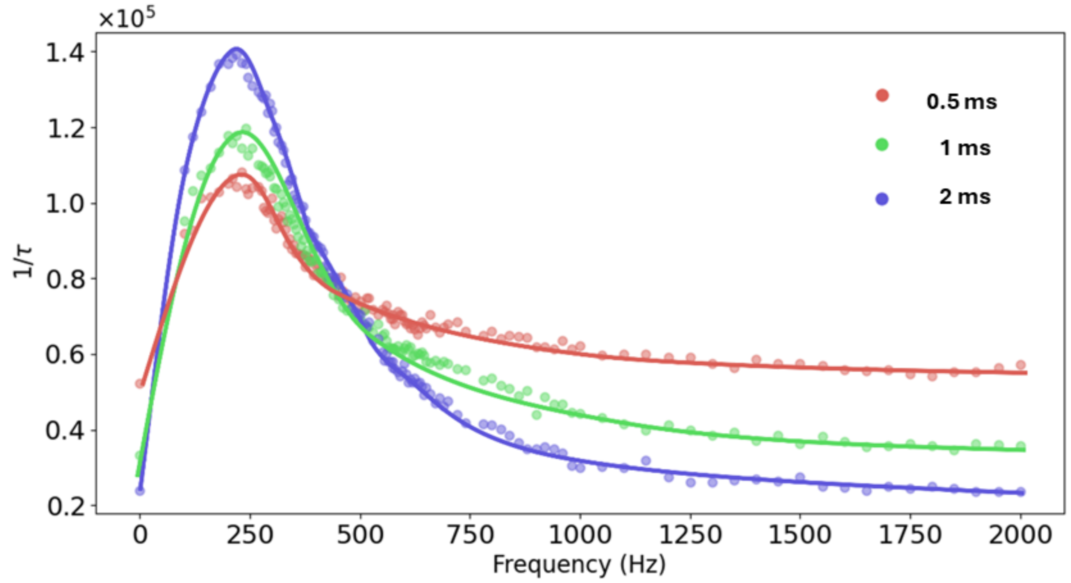

Fig. S1. Comparison of exposure times on resonant curves for homogeneous 0.4% agar phantom. The experiments were conducted using three camera exposure times, 0.5 ms, 1 ms and 2 ms. For all exposure times, the results show the same resonant frequency for τ of 212 Hz. All exposure times produced resonant curves with a peak frequency of 212 Hz. The longest exposure time of 2 ms produced the highest peak value in comparison to the background τ value at 0 Hz, showing the largest signal to noise ratio, and chosen to be the most appropriate exposure time to image the fast dynamics of the propagating surface waves on the sample. Higher exposure times were not tested due to the necessity to keep the acquisition rate above 200 frames per second.

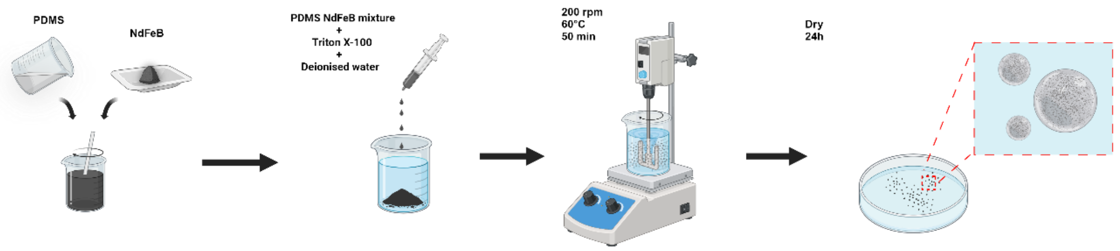

Fig. S2. Methodology to fabricate the microrobots. Uncured PDMS elastomer and NdFeB micro particles were mixed in a beaker by hand. The uncured PDMS NdFeB mixture was degassed for 30 minutes prior to being heated at 70°C for 15 minutes in the vacuum oven to partially cure the elastomer and increase its viscosity. 1ml of elastomer was added dropwise to 100 ml of deionised water and 0.5 ml Triton X-100 solution. The solution was stirred using an overhead mechanical stirrer at 70°C for 2.5 hrs. The particles were removed from the water and dried. Once dry the particles were washed again in deionised water and collected. Schematic produced using BioRender (www.biorender.com).

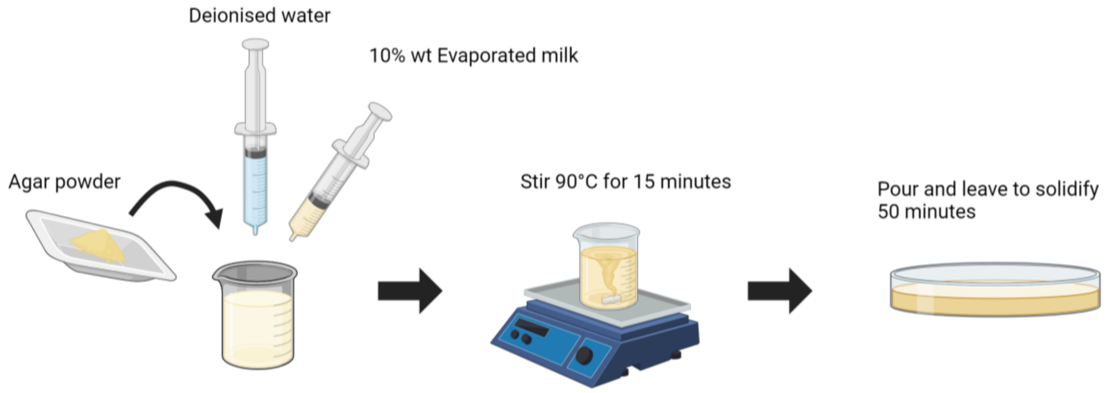

Fig S3 Experimental procedure to fabricate the agar phantoms. Fifty milliliters of deionized water was introduced into a 100 mL Erlenmeyer flask. Agar powder, at concentrations ranging from 0.4% to 1.6% by weight, was introduced into the flask and mixed until dissolved. Additionally, 5 mL of 10% by weight evaporated milk was added to the mixture. The entire mixture was kept under constant stirring at 200 rpm at 90 °C. After thorough mixing and heating, the solution was poured into Petri dishes designated for each sample. The Petri dishes were then left undisturbed at room temperature to allow the mixture to solidify, which took approximately 50 minutes. Once solidified, the gel samples were ready for further use and analysis.

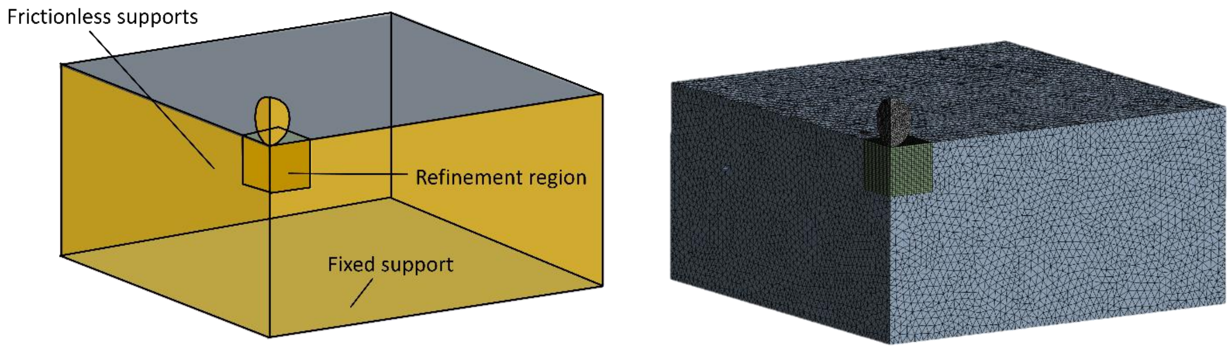

Fig. S4. Finite element simulation setup. To simulate the response to sinusoidal excitation of a microrobot on a tissue sample we conducted Harmonic response in ANSYS 2023 R2 using the harmonic response toolbox. The geometry was configured in a quarter model due to the symmetric nature of a sphere in contact with an elastic half space. The mesh was constructed in a way such that the total model consisted of 123,235 elements. At the contact surface between the microrobot and the top surface of the sample, the mech was refined. The meshing resolution is sufficient to cover the waves propagating through the sample. The boundary conditions were set up with frictionless supports on middle faces to allow for symmetry to be modelled. A fixed support constraint was used on the base. The outer edges of the model were left unsupported. The elastic modulus of each sample was set to be the same as the measured ground truth values and a density of 1000 kg/m^3 a Poisson ratio of 0.5. The Elastic modulus of the microrobot was set as 3 MPa to represent that of hard PDMS and a density of 4000 kg/m^3, calculated based on the ratio of magnetic microrobots to PDMS solution given their respective densities. The frequency sweep was conducted from 0 to 2000 Hz with a maximum harmonic force given as 1E-7 N.

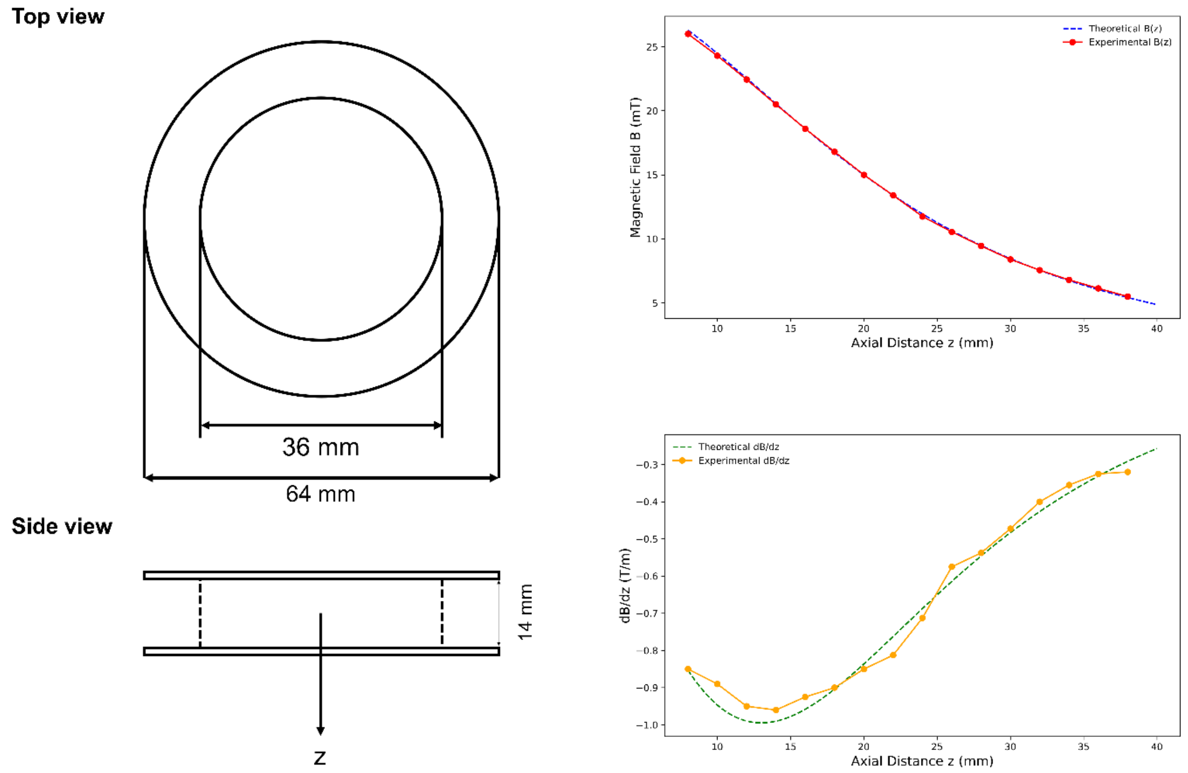

Fig. S5. Schematic of electromagnetic coil. The electromagnetic coil was fabricated by first 3D printing a spool of 36 mm inner diameter and 64 mm outer diameter. The width of the coils was 14 mm. Using an automated coil winding machine, 250 turns of 0.45 mm diameter wire were applied to the spool. To measure the magnetic field, the coil was connected to a DC power supply and supplied with 2A. A calibrated hall sensor attached to a vertical linear optical stage was used to record the values of magnetic field strength (B) in uniform intervals. The magnetic field gradient plot was calculated by differentiating the values of magnetic field strength with respect to axial distance. Both measurements fit well to the theoretical values of magnetic field and magnetic field gradient using the Bio-Savart law for current flowing through a coil of wire.

$$\boldsymbol{B(z)} = \frac{\boldsymbol{\mu_0 N I a^2}}{\boldsymbol{2(a^2 + z^2)^{\frac{3}{2}}}}$$

Where μ_0 is the permeability of free space, N is the number of turns in the coil, a is the radius of the coil, I is the coil current and z is the axial distance from the center of the coil.

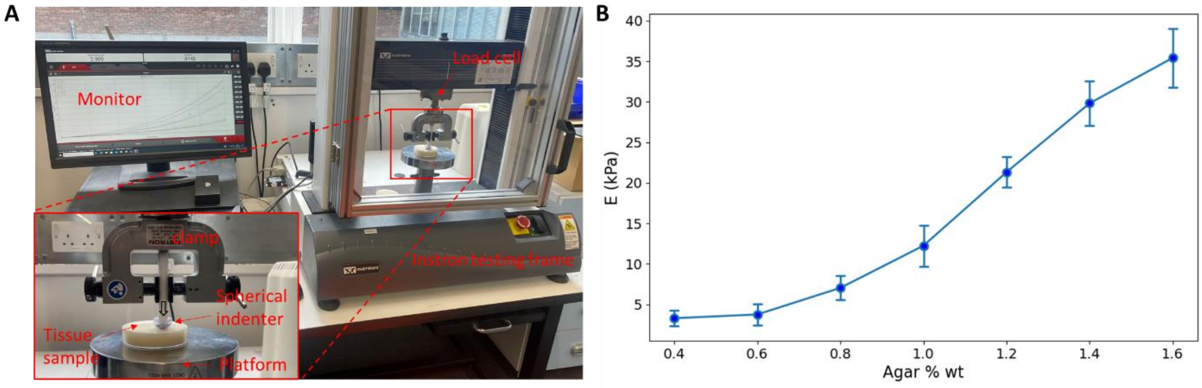

Fig. S6. Methodology to measure the ground truth Young's modulus of homogeneous agar phantoms. The Young's modulus of different tissues was determined using a sphere-plane contact method. The experiment was conducted using a material test frame (Instron, Norwood, MA, USA), as shown in Figure S4. The indenter was made of polyethylene with a diameter of 20 mm and was mounted onto a clamp connected to a load cell. The tissues were evenly filled into a petri dish with a diameter of 90 mm, placed directly beneath the indenter on the platform of the testing machine. To simulate tissue stiffness corresponding to different cancer stages, tissues were fabricated using various ratios of agar powder to water (Special Ingredients, UK). Seven different stiffness levels of tissues were prepared. The preparation process was identical to that shown in Figure S2. Specifically, the mixing weight percentages of agar powder to water were, 0.4%, 0.6%, 0.8%, 1.0%, 1.2%, 1.4% and 1.6%, each with 10% wt evaporated milk added.

During the testing process, a lubricating gel (Loovara, Germany) was applied around the indenter to minimize friction effects. To reduce experimental errors caused by tissue fabrication inconsistencies, 4 different locations on each tissue sample were measured, with each location being tested 3 times. Adequate time intervals were allowed between each measurement to ensure that the tissue returned to its initial state. The high-precision displacement and force sensors within Instron machine recorded the deformation (D) and the interaction force (F) in the vertical direction. The Young's modulus (E) of the tissue was then calculated using the Hertz contact model, given by the formula:

$$E = \frac{3}{4} \cdot R^{-\frac{1}{2}} \cdot (1 - \nu^2) \cdot D^{-\frac{3}{2}} \cdot F$$

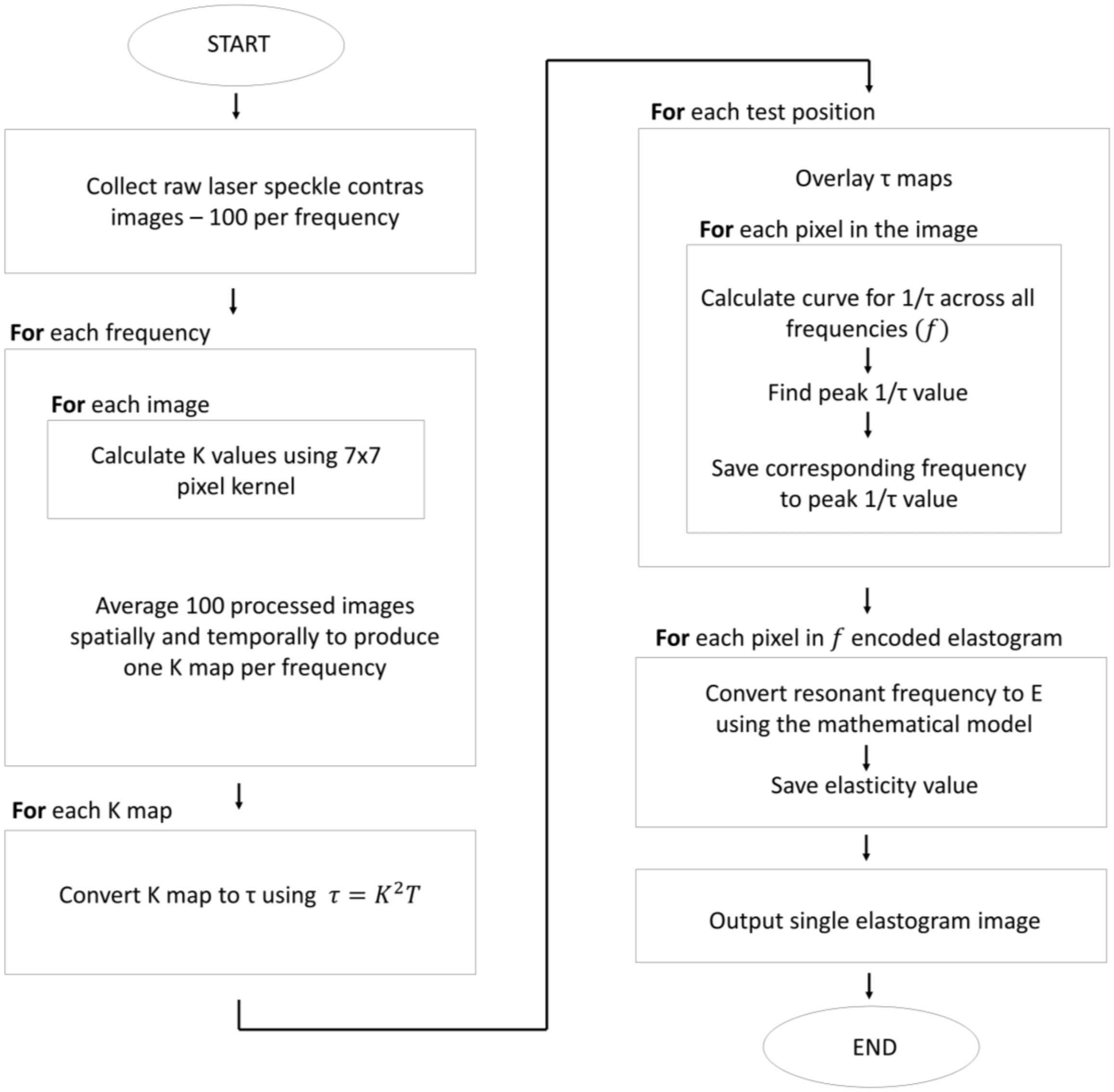

Fig. S7. Image processing method to generate elastograms images. In the laser speckle contrast imaging (LSCI) analysis, we collected raw speckle contrast images at various frequencies, acquiring 100 images per frequency step. Each image underwent processing to calculate the K laser speckle contrast values $K = \frac{\sigma}{\langle I \rangle}$ using a predefined equation within a 7x7 pixel kernel. This process yielded 100 processed images, which were then averaged spatially and temporally to produce a single K map for each frequency. The K map was subsequently converted to a map of τ values using the relation $\tau = K^2T$ where T is the exposure time. This procedure was repeated for each frequency step at each test position. Next, we overlaid all tau maps corresponding to each test position and iteratively analysed each pixel. For every pixel, we plotted the curve of 1 /τ across the frequency values to identify the peak value, representing the resonance frequency. This peak frequency was recorded, resulting in a resonant frequency-encoded elastogram. Next, we converted the resonant frequency of each

pixel into elasticity values using the mathematical model, resulting in a single elastogram that maps the elasticity distribution across the sample. All analysis was conducted in MATLAB 2023b (MathWorks, USA).

Movie S1.

An example of a speckle movie of the microrobot being positioned on the sample by rolling across the surface to the points where the frequency sweeps are conducted.
